# Supplementary material for: Insights into cultural and compliance challenges in type 2 diabetes care: A qualitative study of Moroccan and Belgian patients in Belgium
Source: PLoS One. 2024 Nov 14;19(11):e0310800. doi: 10.1371/journal.pone.0310800 (PMC11563466; doi:10.1371/journal.pone.0310800)
Supplement: S1 Table — (DOCX) [file pone.0310800.s001.docx]

**S1 Table Themes, subthemes and illustrative quotes.**

Themes are indicated in capitals, subthemes in bold, and codes in italic. Illustrative quotes are translated verbatim. (B=Belgian descent, M=Moroccan descent, F=Female, M=Male)

| **Theme** | **Illustrative quotes** |
| --- | --- |
| BELIEFS ABOUT DIABETES |  |
| **Diabetes as disease** |  |
| *Insufficiently informed* | Q1. I don't know what to think about it. (...) I often suffer from hypoglycaemia. I don't know what to do then. I have called many times, but I don't get any advice. (B7F)  Q2. I didn't actually get that much information. After the consultation, I got a paper to go to the dietician and basically that's all (M3F). |
| *Not enough preventive information* | Q3. Of course I had to have known 10 years ago what I know now. Yes then I shouldn't have come here. Because then I would have moved more and been more mindful of my diet. And then it might not have come this far. (B1M)  Q4. Yes it is a question of education! What are they going to give Moroccans as good advice? They hardly know anything about it! But it is not their fault! There are many who cannot read or write. (M2M)  Q5. More information needs to be given. To tell them that it may well be God who has given us the disease. But it is up to us to control the disease! (M6F) |
| *Unhealthy lifestyle as a cause of diabetes* | Q6. In my case, it must be my Burgundian lifestyle. (B2M)  Q7. Because I wasn't following a proper diet. That's very simple! I ate everything, the cakes just flew in. For me, that was not something I thought about. And I was also drinking and of course smoking, which is not really conducive either. (M2M) |
| *Hereditary factor of diabetes* | Q8. Why I got diabetes I don't know. I think it will probably have to do with it being hereditary. (B8F)  Q9. Heredity also plays a role. It's a bit like bingo in fact. I do and my other sisters don't. (M8F) |
| *Stressful or emotional event was associated with getting diabetes* | Q10. I contracted diabetes because of a car accident. I then experienced an emotional shock (M2M). |
| *Diabetes is God's will* | Q11. It is God's decision that I get diabetes and I accept it because I have no other choice. (M9M) |
| *Nature of diabetes disease* | Q12. I hope it will end. But it is difficult. It won't. Somehow you take the medicine but the diabetes is still there. It's always there. (M1M)  Q13. It is not the end of the world, there is treatment, there are different paths you can take. (M7F) |
| *A tiring and restrictive disease* | Q14. Tiring to measure all the time and you don't see the results. (M1M) |
| *Diabetes is not an exception in the Moroccan community* | Q15. I have no fear of the disease. My father, for example, he is almost 93 and he is diabetic. So is my mother and she is 85. So I have no fear! (M3F)  Q16. It is also just assumed that the disease diabetes is something normal within our community! (M5F) |
| **Diabetes and medication** |  |
| *Confidence in doctor and focus on results* | Q17. We switched to that Galvus. (...) The reason why will probably have to do with the blood results heh. That they are not what the doctor expected. I am being informed well enough. For me it's mainly the result that counts. (B1M) |
| *Wanting to swap pricking for sensor* | Q18. I have already talked about this. If there is a way to get a sensor or something that measures blood without needles. They said it exists but you have to pay for it and it's expensive. I can't afford it. And I regret that. (M1M) |
| *Impact of medication on personal life* | Q19. It changes a lot for me, for example the insulin you have to inject. Once you do that it changes your life. (...) You have to remember to do it and then wait and then eat. (B3F)  Q20. No it doesn't really affect the course of my day. (M2M) |
| *Use of seeds to control blood sugar levels* | Q21. Those seeds, that's what we eat during our Moroccan meals, that lowers sugar levels. (...) That gives you strength and those seeds filter the sugar levels, if you keep this up for one week, you learn to eat it and your sugar levels drop. That is very good! (M7F) |
| **Diabetes and lifestyle** |  |
| *Avoiding sweets* | Q22. You have to think about many things. You are not allowed to eat many things and you have to watch what you do all the time. You have to watch the amount of sugar you eat, you should not eat too many sweets. (M5F) |
| *Additional efforts for physical activity* | Q23. I try to exercise a bit more by walking the dog. And I do that mostly on weekends now. (B2M) |
| *Not necessary to adjust eating habits* | Q24. No I don't think it is necessary to change my eating habits as a function of my illness. (M5F)  Q25. I think that doesn't bother me I still continue to appreciate Moroccan cuisine which is very rich. (M8F) |
| *Compensate with additional vegetables* | Q26. Instead of a whole steak, I only take half. Fries instead of frying them in oil, well now I fry them in a fryer without oil. And I make up for it with tomatoes and cucumbers. (M4M) |
| *Not wanting to avoid certain foods for the sake of pleasure* | Q27. Cake I can eat now too lol. I'm not always going to avoid eating sweet things for the sake of illness because then I get sad! But from that one, they do take away my pleasure. I'll keep it in mind. (B3F) |
| *Difference between generations* | Q28. There does seem to be a difference in lifestyle between generations. In general, the older generations are more used to traditional Moroccan cuisine. And the younger ones are more flexible and eat a more European cuisine (M8F). |
| DOCTOR-PATIENT RELATIONSHIP |  |
| *Passive attitude towards doctors* | Q29. I listen to what they say. In terms of participation, I haven't made any demands yet. I assume they know what they are doing. (B2M) |
| *Actively seeking information yourself* | Q30. There is never a moment where I don't actually understand what the doctor is saying. And if I wouldn't understand something, I look it up in a dictionary or a scientific book at home. (M2M) |
| *Trusting GP regarding diabetes* | Q31. I just go to my GP for minor treatments, but for my diabetes I don't trust him. (M9M)  Q32. I don't really know what to expect from a GP specifically about that diabetes. (...) They have to be somewhat at home in all markets but do they really have to be right in my case, does he really have to sort that out? That's up to the diabetologists. (B1M) |
| *Clarity of information* | Q33 Q5. He is French-speaking but can speak Dutch well and for medical matters you like to have information in your own language. It depends from hospital to hospital. You do like to have your own language when it comes to medical matters. Language does a lot because you understand the difficult terms better. (A3V) |
| *Information within hospital may be more comprehensive* | Q34. I then eat a sweet, so my values go back up. But I just don't know. And I am not being helped. This scares me, and the doctor says nothing about this. My blood values are good then according to him, he says carry on. I am not being taken seriously. (B7F)  Q35. Yes, he did explain the general causes but specifically for me, he didn't really discuss the causes. I can't remember anyway. (M5F) |
| *Not being honest with diabetologist* | Q36. I also lied to doctors about my eating style. The doctor then suggested starting insulin because the values kept rising. (M6F) |
| BARRIERS AND FACILITATORS |  |
| **External barriers** |  |
| *Social events lead to bad eating habits* | Q37. Especially at weekends heh! So we have a mobile home and - the social life is a bit less now with corona, but before that we would be getting together at weekends, barbecue at that, other weekends at someone else's. Then it's hard to say no. (B2M)  Q38. They don't want to change their eating style. They don't easily move away from their traditional pastries and accompanying coffee or tea. They also eat bread with all dishes. And all sauces are also very greasy. I try to eat varied food. They stick to their traditional diet. (M6F) |
| *Dominance of male perspectives in Moroccan culture* | Q39. Women enjoy hardly anything in terms of education outside the household. For example, if I buy something I at least read on the packaging what is effectively in that product. And they often can't even read so they don't know all that. But they just get on with it! (M2M)  Q40. Women are usually submissive, the man is usually the boss in the house. The woman usually has to listen to the man's wishes, their life is more difficult. (...) And this can then have an effect on bad living habits. (M6F) |
| *Lack of support leads to poor eating habits* | Q41. You have to leave a lot behind. You also have to have the character and support of the people around who help you to do that. Having support helps a lot. That you are not allowed to snack anymore. (...) My husband likes to eat biscuits for breakfast. I dare to eat two pastries then. (B3F) |
| *Lack of support leads to low physical activity* | Q42. I am single and that means I get little motivation to exercise. And then it's easy to fall back on things like idleness. A partner pulls you along. Friends too! (B5M) |
| *COVID stops participants from moving* | Q43. I was enrolled in a Basic Fit club. Now I don't go anymore because we can't go. This corona thing, it crushed us. It is very difficult. (M1M) |
| *Limited influence of physician on adherence* | Q44. He has an influence: yes and no. He asks me to do a lot of things and then sometimes I think when I want to go and eat a burger I am not going to do it anyway to satisfy the doctor. But sometimes I also think the opposite and really don't care what the doctor is going to say. And then I eat less healthy (M8F).  Q45. But the doctor told me in the beginning when I started treatment: your pancreas may start making insulin again. But unfortunately I am not steady in my treatment. (M1M) |
| *Practicalities lead to medication being taken incorrectly* | Q46. But yes if you are somewhere external on the job then yes, normally you would go somewhere in a restaurant to eat something and then you could do that there and now it is a sandwich in the car. Often then with a colleague next to you. Then it's a bit trickier. (B2M)  Q47. If you have several rash marks on the belly in a certain place, then you say you are not going to inject there today and rather you are going to wait for a while and then just continue (without injecting). And that has already happened to me. (M5M) |
| *Ramadan participation leads to incorrect medication intake* | Q48. My doctor knows that I am participating in Ramadan. I know how to deal with the disease and how to get through Ramadan. I don't take my medication but if I feel really bad I do eat because the body feels it when the level is too high or too low and then I take my medication. As a diabetic you just feel that. And if it's really too low then I take them (M9M). |
| *Lower adherence to therapy in winter months* | Q49. It is to say, every year in winter there was an increase. And in fact, it makes sense because in winter you get out less and you're not moving as much. And then again, I like sweets, so then it was about 2 or 3 tangerines every night. (B1M)  Q50. There is a difference. I would say it's harder in winter. Hours go by faster and you can forget about it more often. In summer, it's okay. In summer you are in a better mood. (M1M) |
| BARRIERS AND FACILITATORS |  |
| **Internal barriers** |  |
| *Taking medication incorrectly due to forgetfulness* | Q51. The quantity and fatigue play a role in that. Then when I take medication it can happen that I just forget to take a certain medication because I have so many. (M5F)  Q52. At 22h15, the long-term insulin was then injected. And I did have problems with that. Sometimes I was asleep before I got there (B5M). |
| *Adjusting medication dose based on meal* | Q53. Sometimes when I find I'm going to eat something that doesn't have too many sugars I decide not to take my medication myself. Like when I eat a salad with chicken, for example. Then I don't take them. (M8F)  Q54. Everything depends on what I eat, if I feel bad I eat more sugars, then I inject a few more units. (M6F) |
| *Wrong attitude towards treatment* | Q55. In the beginning, I was a bit sceptical, I didn't believe in it very much. I thought it was a simple disease like a cold with no consequences. (...) That's why I didn't have much interest in taking my medicines. When I forgot to take them, I didn't think it was serious. (B8F)  Q56. One day I do it, two days I don't and that's it. I tell myself it's always the same. I see the results. So there's no point. (M1M).  Q57. And I feel that the medication also weakens me somehow. I really have to take a lot of medication during the day and it's really not that easy! (M5F) |
| *Poor compliance due to stress* | Q58. As soon as I have a moment of mental difficulty and go into a slump, I spend less attention on the disease. (M6F)  Q59. The stress and on an emotional level it's also often unstable. And that also reflects in the diet these flare-ups. (M5F) |
| *Intrinsic motivation to exercise* | Q60. I used to but not anymore. I used to be an indoor football referee, Sunday afternoons I was a linesman. So I was quite active. (B5M) |
| **External facilitators** |  |
| *Support from social environment* | Q61. My wife was there at the time and I asked her for help because I wouldn't be able to overcome this on my own. (...) My wife also makes sure I take everything right! (M2M)  Q62. Yes, I can't complain. Really. I have four fantastic children at that point. Now they take turns (in helping me). (B6F) |
| *Good medical professional support* | Q63. I think if I had been another patient they would have given her insulin, but when I come to see her she scares me a little bit because she asks me to lower my weight, because if she checks my weight and she sees that I have gained weight she threatens to give me insulin. And I love this doctor because she goes out of her way not to give me insulin. (M9M) |
| *Not participating in Ramadan is conform religious rules* | Q64. Since I am diabetic, I am considered someone who is sick. So I have the right to eat. So I do not participate in Ramadan. So I don't adjust my medication either. (M3F) |
| *Maintaining healthy lifestyle easier through retirement* | Q65. And I think if you want to adapt your life to it, your way of life... I realise that for someone who is in an active period or in an active life, it is more difficult as for someone who has already evolved. (B6F) |
| **Internal facilitators** |  |
| *Day structure helps medication intake* | Q66. I have a box where my medications are in and I know what to take in the morning, afternoon and evening and I have got used to that. (M9M)  Q67. You get used to that hey. That becomes a routine in the morning, I get up 15 minutes early for that. I put them in a bag from the supermarket here. Yes every morning I just take those. The ones from the evening are on my night table and before going to bed I take them. I actually have little problem taking that. I rarely skip that. (B2M) |
| *Wishing to improve* | Q68. I know I have to take care of myself and that is a rule of thumb and I have to take them to take care of myself. (B8F)  Q69. Yes, I am obliged to take the medication. If I were to take my medication one day and not the other day I am playing with my own health. (M3F) |
| *Taking medication due to fear* | Q70. Yes but honestly I do have some fear, so I do take the medication. (M7F) |
| *Self-control* | Q71. But I have to be disciplined and that's what I did. I absolutely must not cross the red line as they say! I have to be disciplined! (M4M)  Q72. Steadfastness. You must not let yourself go. And that is not always easy. For example, when injecting when you don't feel like injecting. (B5M) |
