# Supplementary material for: Insights into cultural and compliance challenges in type 2 diabetes care: A qualitative study of Moroccan and Belgian patients in Belgium
Source: PLoS One. 2024 Nov 14;19(11):e0310800. doi: 10.1371/journal.pone.0310800 (PMC11563466; doi:10.1371/journal.pone.0310800)
Supplement: Attachment — Submitted filename: response to reviewers.docx [file pone.0310800.s002.docx]

Dear Dr. Belkadi,

Thank you for the opportunity to revise our manuscript. We have addressed the reviewer's points and your comments regarding the journal's requirements in the table below.

We hope our revised manuscript meets the standards.

With kind regards and appreciation for your voluntary work as editor,

Dr. Stefaan Six (on behalf of all authors)

| Comments | Author reply |
| --- | --- |
| Editor |  |
| 1. When submitting your revision, we need you to address these additional requirements.  Please ensure that your manuscript meets PLOS ONE's style requirements, including those for file naming. The PLOS ONE style templates can be found at  https://journals.plos.org/plosone/s/file?id=wjVg/PLOSOne_formatting_sample_main_body.pdf and  https://journals.plos.org/plosone/s/file?id=ba62/PLOSOne_formatting_sample_title_authors_affiliations.pdf | We have adjusted the manuscript so it complies with all PLOS ONE’s style requirements. |
| 2. We note that your Data Availability Statement is currently as follows: All relevant data are within the manuscript and its Supporting Information files.  Please confirm at this time whether or not your submission contains all raw data required to replicate the results of your study. Authors must share the “minimal data set” for their submission. PLOS defines the minimal data set to consist of the data required to replicate all study findings reported in the article, as well as related metadata and methods (<https://journals.plos.org/plosone/s/data-availability#loc-minimal-data-set-definition>).  For example, authors should submit the following data:  - The values behind the means, standard deviations and other measures reported; - The values used to build graphs; - The points extracted from images for analysis.  Authors do not need to submit their entire data set if only a portion of the data was used in the reported study.  If your submission does not contain these data, please either upload them as Supporting Information files or deposit them to a stable, public repository and provide us with the relevant URLs, DOIs, or accession numbers. For a list of recommended repositories, please see <https://journals.plos.org/plosone/s/recommended-repositories>.  If there are ethical or legal restrictions on sharing a de-identified data set, please explain them in detail (e.g., data contain potentially sensitive information, data are owned by a third-party organization, etc.) and who has imposed them (e.g., an ethics committee). Please also provide contact information for a data access committee, ethics committee, or other institutional body to which data requests may be sent. If data are owned by a third party, please indicate how others may request data access. | We confirm that the Data Availability Statement is correct. |
| 3. PLOS ONE now requires that authors provide the original uncropped and unadjusted images underlying all blot or gel results reported in a submission’s figures or Supporting Information files. This policy and the journal’s other requirements for blot/gel reporting and figure preparation are described in detail at <https://journals.plos.org/plosone/s/figures#loc-blot-and-gel-reporting-requirements> and <https://journals.plos.org/plosone/s/figures#loc-preparing-figures-from-image-files>. When you submit your revised manuscript, please ensure that your figures adhere fully to these guidelines and provide the original underlying images for all blot or gel data reported in your submission. See the following link for instructions on providing the original image data: <https://journals.plos.org/plosone/s/figures#loc-original-images-for-blots-and-gels>.    In your cover letter, please note whether your blot/gel image data are in Supporting Information or posted at a public data repository, provide the repository URL if relevant, and provide specific details as to which raw blot/gel images, if any, are not available. Email us at [plosone@plos.org](mailto:plosone@plos.org) if you have any questions. | NA (no images in the manuscript) |
| 4. Please review your reference list to ensure that it is complete and correct. If you have cited papers that have been retracted, please include the rationale for doing so in the manuscript text, or remove these references and replace them with relevant current references. Any changes to the reference list should be mentioned in the rebuttal letter that accompanies your revised manuscript. If you need to cite a retracted article, indicate the article’s retracted status in the References list and also include a citation and full reference for the retraction notice. | We have reviewed the reference list for completeness and correctness. |
| Reviewer 1 |  |
| 1. The text can replace the reference to “God” with religious belief. | Thank you for your comment. We have replaced all references to “God” with a reference to religious belief. |
| 2. Did authors find any difference in adherence between first-generation Moroccans and second- and third-generation Moroccans? | In our study, the primary intergenerational difference observed was in dietary habits, with older generations appearing to adhere more strictly to traditional Moroccan cuisine. This finding is detailed in the Results section under the theme “Beliefs about diabetes”. However, the sample included only one second-generation and one third-generation Moroccan immigrant. Due to this limited representation, we did not analyze differences in medication adherence between the generations within the Moroccan group. The small sample size would not have provided meaningful conclusions about generational differences in adherence. Instead, our focus was on the broader cultural and compliance challenges between Belgian and Moroccan groups. Future quantitative research with a larger sample size is necessary to address the valuable remark of the reviewer, i.e. to explore intergenerational differences in medication adherence among Moroccan immigrants more comprehensively. |
